# Supplementary material for: DMRforPairs: identifying Differentially Methylated Regions between unique samples using array based methylation profiles
Source: BMC Bioinformatics. 2014 May 15;15:141. doi: 10.1186/1471-2105-15-141 (PMC4046028; doi:10.1186/1471-2105-15-141)
Supplement: Additional file 2 — DMRforPairs output for the comparison of A431-MCF7 and NA17018-NA17105. Please start from the HTML files in each folder. Available via the BMC Bioinformatics website. [file 1471-2105-15-141-S2.zip › 1394847754114233_MOESM2_ESM/A431_MCF7/figures/18472.pdf]

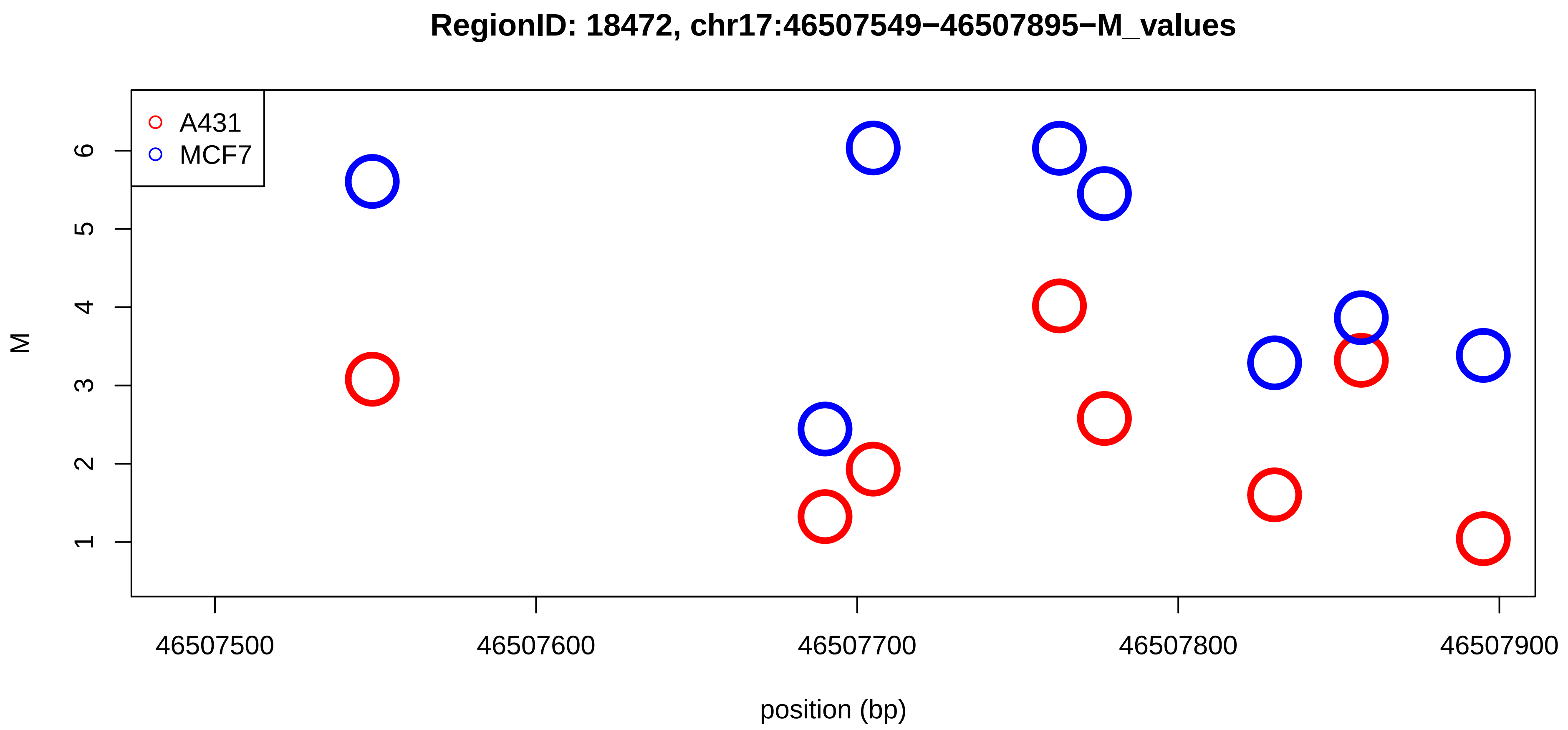

RegionID: 18472, chr17:46507549−46507895−M_values
A431
MCF7
M
1
2
3
4
5
6
46507500
46507600
46507700
46507800
46507900
position (bp)

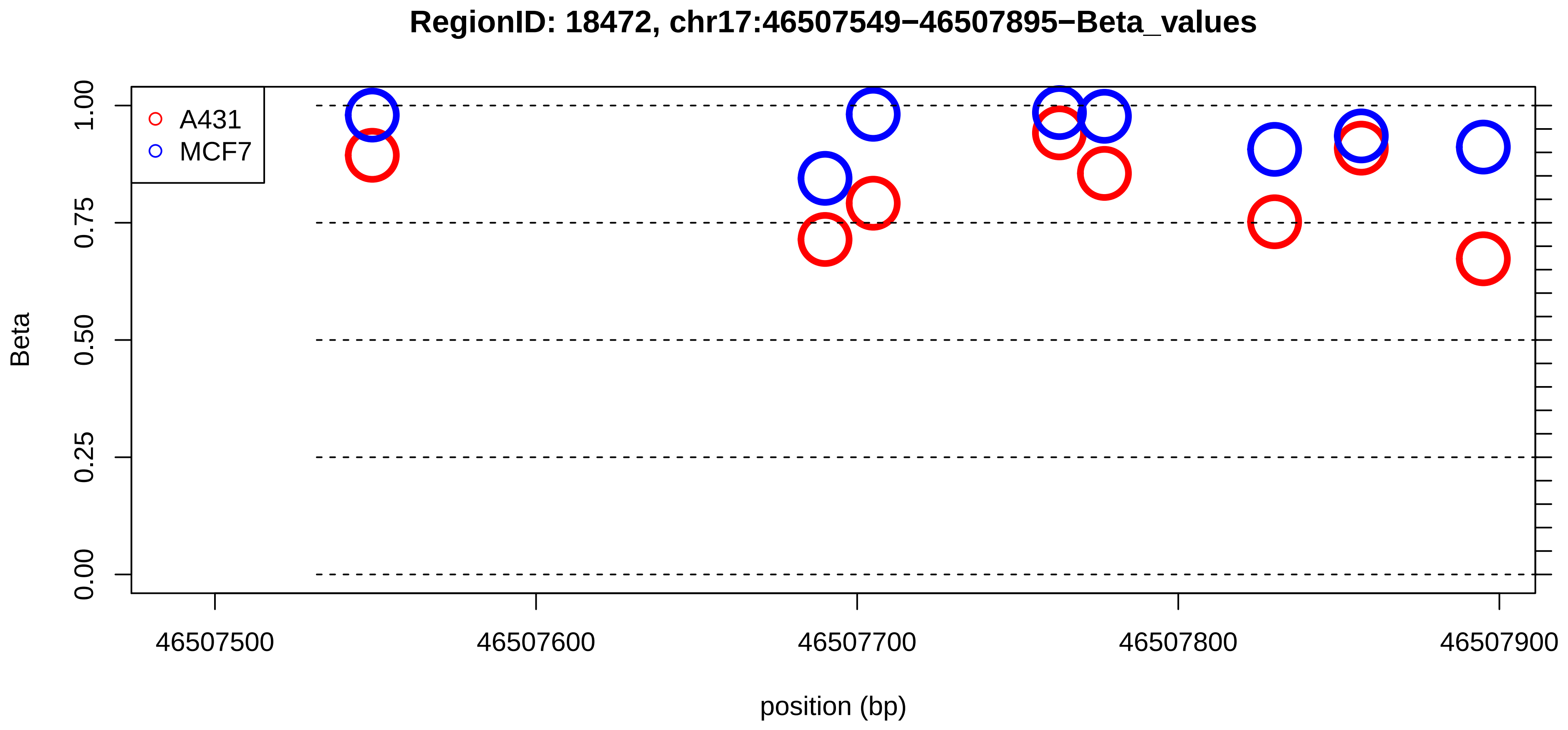

RegionID: 18472, chr17:46507549−46507895−Beta_values
A431
MCF7
Beta
0.00
0.25
0.50
0.75
1.00
46507500
46507600
46507700
46507800
46507900
position (bp)

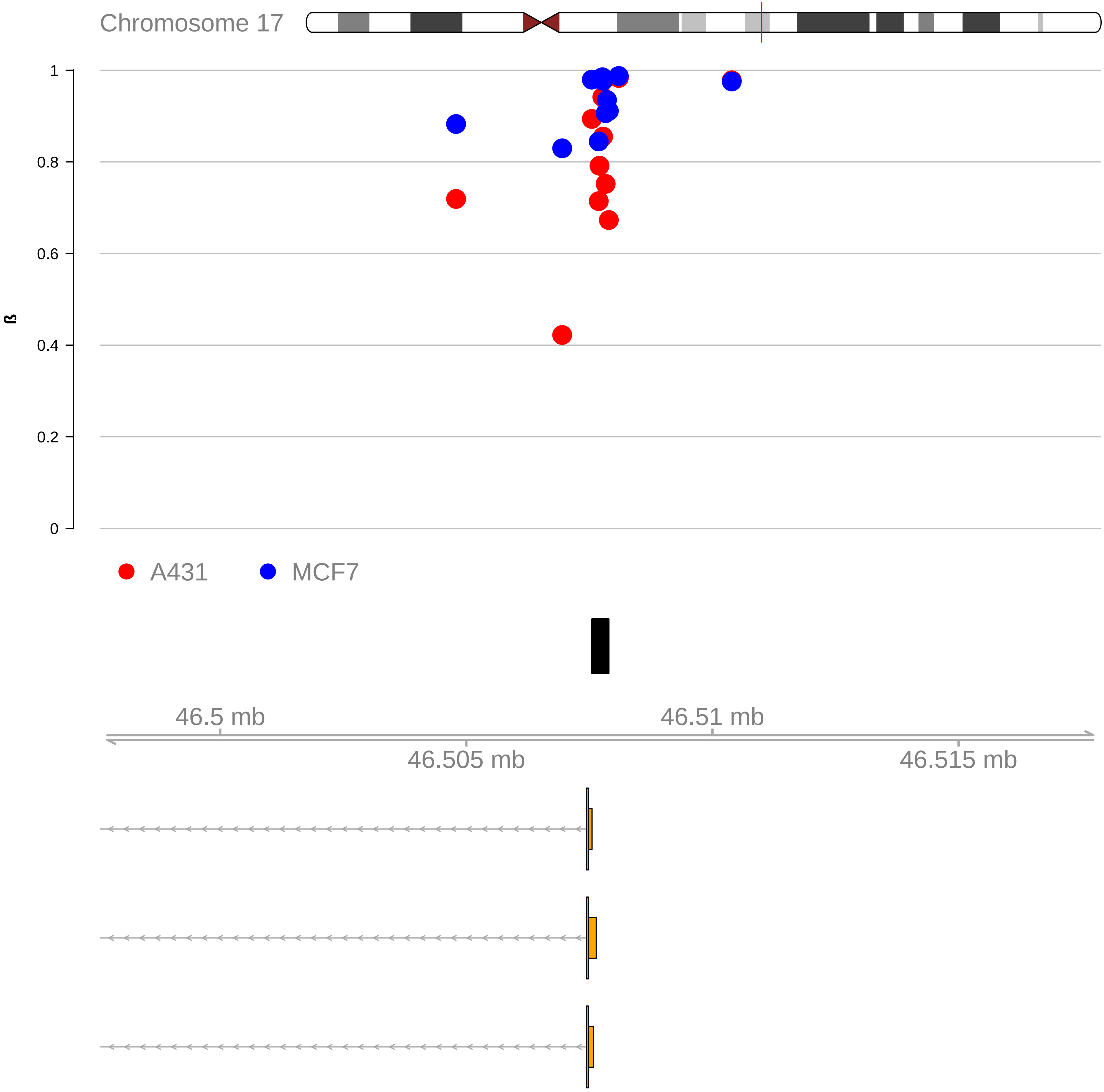
Chromosome 17
1
0.8
0.6
ß
0.4
0.2
0
A431
MCF7
46.5 mb
46.51 mb
46.505 mb
46.515 mb
